# The epidemiology of the most frequent cancers in Poland in 2015–2021 and the impact of the COVID-19 pandemic on cancer incidence

**DOI:** 10.1186/s13690-024-01277-6

**Published:** 2024-04-15

**Authors:** Piotr Choręza, Aleksander Jerzy Owczarek, Wacław Kruk, Jerzy Chudek

**Affiliations:** 1 Department of Medical Statistics, Medical University of Silesia in Katowice, Ostrogórska 30 Street, 41-200 Sosnowiec, Poland; 2 Health Promotion and Obesity Management Unit, Department of Pathophysiology, Medical University of Silesia in Katowice, Katowice, Poland; 3 Department of Nursing and Public Health, University of Rzeszów, Rzeszów, Poland; 4 Department of Internal Medicine and Oncological Chemotherapy, Medical University of Silesia in Katowice, Katowice, Poland

**Keywords:** Cancer, Epidemiology, COVID-19, Poland

## Abstract

**Background:**

The late diagnosis, despite the improving availability and accessibility of diagnostic procedures during the last decade in Poland and cooperation between specialist cancer centres, remains an unsolved problem. Moreover, the accessibility to healthcare resources and diagnostic procedures has been drastically reduced because of the COVID-19 pandemic in 2019–2020. The study aimed to present the epidemiology of the most frequent cancers diagnosed in Poland as well as the impact of the COVID-19 pandemic on cancers’ incidence.

**Methods:**

Depersonalized, epidemiological data was obtained from the National Health Fund of Poland. In this retrospective study, the epidemiological analysis was performed and divided into subregions, according to patients’ domicile. For each of the subregions, we have calculated the incidence rate per 100,000 standardized to the European Standard Population 2013. The time points of providing the first healthcare service were considered as the time of cancer diagnosis.

**Results:**

In the 2015–2019 period, before the COVID-19 pandemic occurred, the nationwide incidence of analysed cancers remained stable or slightly decreased (as the lung cancer). Simultaneusly, during the same period the prevalence of the prostate cancer has increased and the large differences between subregions with the least and the highest incidence were observed. Subsequently, the incidences of all analyzed cancers decreased in 2020, compared to the period before the COVID-19 pandemic occurred. Then, in 2021 a disproportionate increase in cancers’ incidence rates was noted.

**Conclusions:**

Our results show a significant decrease in the incidence rate of the most frequent cancers diagnosed in Poland in 2020 compared to 2019. Subsequently, in 2021 the increase of the incidence ratios was noted, most likely due to the gradual reduction of epidemic restrictions.

**Supplementary Information:**

The online version contains supplementary material available at 10.1186/s13690-024-01277-6.

**Table:** 

Text box 1 Contributions to the literature
• The assessment of the effect of the COVID-19 pandemic, and the cancer burden on the healthcare system in Poland.
• Many patients whose cancer diagnosis and beginning of treatment were delayed, because of pandemic restrictions and inaccessibility of healthcare services, could be diagnosed in a more advanced stage.
• The increased burden of the most frequent cancers diagnosed in 2021, compared to 2020 indicates a gap in cancer diagnosis, the regional and nationwide screening programs should be more promoted to increase their utilization to enable diagnosis at the earliest stage.

## Background

European population remains only 9% of the world population but it accounts for about 25% of cancer incidence worldwide [1]. Because of the aging of European societies, an increase in the cancer burden in the next years is expected. In 17 European countries, cancer ranked first ahead of cardiovascular diseases as the leading cause of death [2]. According to current estimations, by the end of 2040 the worldwide cancer burden will have achieved 28.4 million incidences, so fighting cancer becomes the one of the most important tasks for the public health of the 21st century [3].

Still late diagnosis, despite improving availability and accessibility of diagnostic procedures during the last decade in Poland and cooperation between specialist cancer centers, remains an unsolved problem.

Cancer remains the second cause of premature death in Poland after cardiovascular diseases. According to the register data, the most frequent cancers diagnosed in Poland in 2020 among women were: breast cancer (C50, according to the International Classification of Diseases 10th revision– ICD-10 classification), lung and bronchus cancer (C34), endometrial cancer (C54), and among men it were: prostate cancer (C61), lung and bronchus cancer (C34) and colon cancer (C18) [4]. Those findings correspond with our previous study concerning two Polish regions: the Silesian and Subcarpatian provinces [5].

At the end of 2019, a new coronavirus SARS-CoV2 causing the COVID-19 disease, occurred in Wuhan, in the Hubei province of China [6]. A new ssRNA virus was similar to the SARS and MERS that had affected the epidemic in 2002 and 2012 respectively [7]. In a few months, it has become the most fundamental problem for public health worldwide. On March 11^th,^ the World Health Organization announced the pandemic that has affected the daily life of ordinary people, the economy as well as the organization and availability of healthcare resources and services [6].

The access to healthcare resources and diagnostic procedures was drastically reduced because of the COVID-19 pandemic in 2019–2020. This stormy situation for the world order and public health politics resulted in the engagement of healthcare resources and staff in the fight against the COVID-19 pandemic. Therefore, the limitation of scheduled healthcare services and impediments in contact with medical doctors, including general practitioners was observed.

The study aimed to present the epidemiology of the most frequent cancers being one of the greatest challenges for the public health and the national healthcare system, according to different geographical districts. Moreover, the analyses covering a period of time both prior to and during the COVID-19 pandemic enable the assessment of the impact of the pandemic and the introduced restrictions as well as the involvement of health care resources for fighting the pandemic on the epidemiology of the most frequent cancers diagnosed in Poland.

### Country´s characteristics

Poland is one of the countries that underwent a structural transformation at the turn of the years the 1980s and 1990s. The economic crisis associated with the fall of communism has affected the healthcare system’s condition. Those changes were multi-faceted. The transition from a centrally controlled to a free-market economy has resulted in an increase in the number of medical services provided by private entities, often financed by the patients. On the other hand, the difficult economic situation resulted in drastic restrictions on investments in the public healthcare sector. At the beginning of the 21st century, some reforms regarding the social security system and the mechanisms of healthcare financing have been introduced, but the effects are unsatisfactory.

Poland remains among the European countries spending the least funds on healthcare. According to the Eurostat data, it was only 6.49% of the gross domestic product (GDP) in 2020. This score is significantly below the EU average (10.9%) and almost twice below spending in Germany (12.82%). This along with the shortage of medical staff causes limitations in the accessibility and availability of healthcare resources and services with their consequences.

**Fig. 1:**
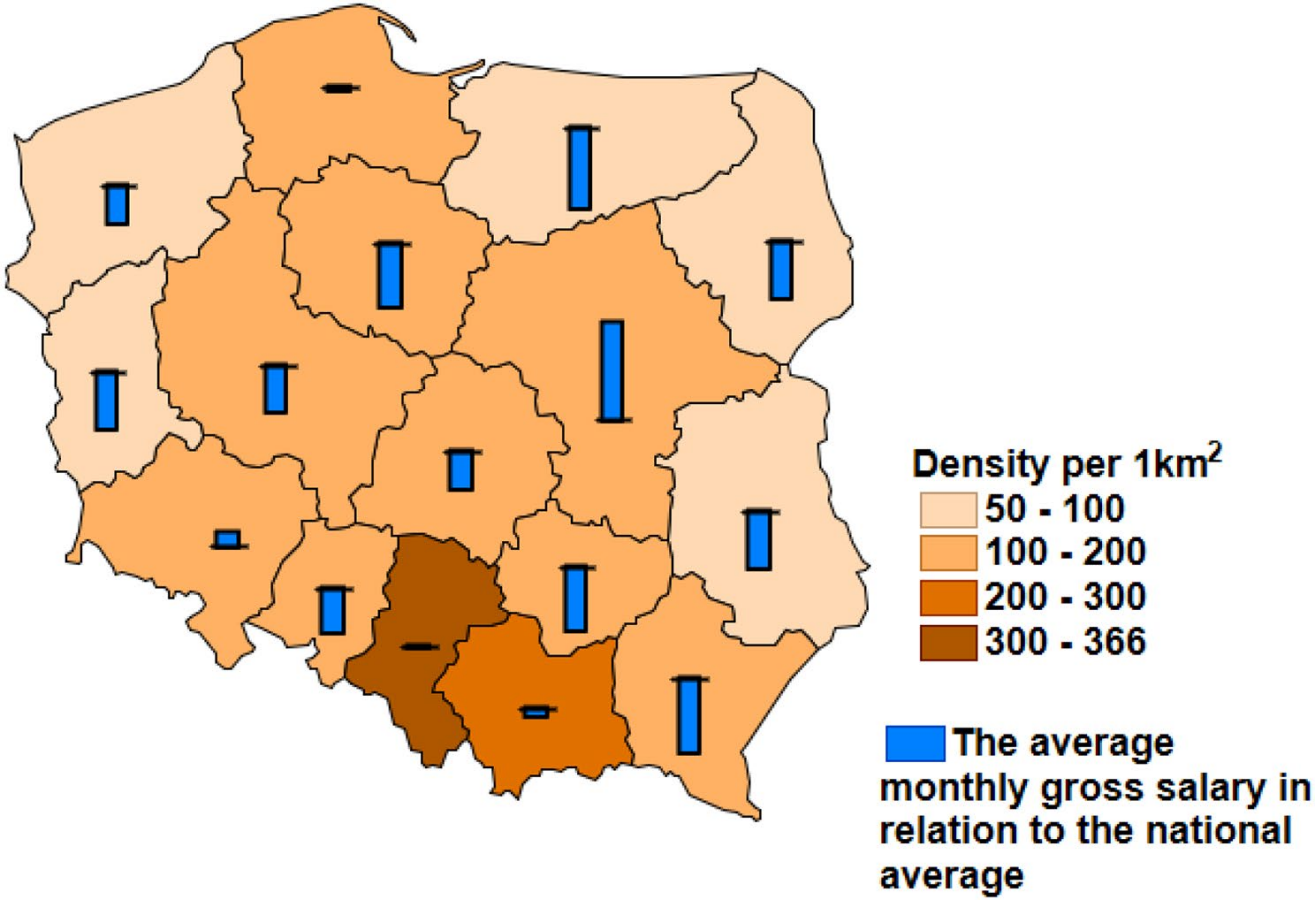
The average density per 1 km^2^ in each of the Polish regions in 2019. Bars present the ratio of the average monthly salary to the national average (presented as horizontal lines)

**Fig. 2:**
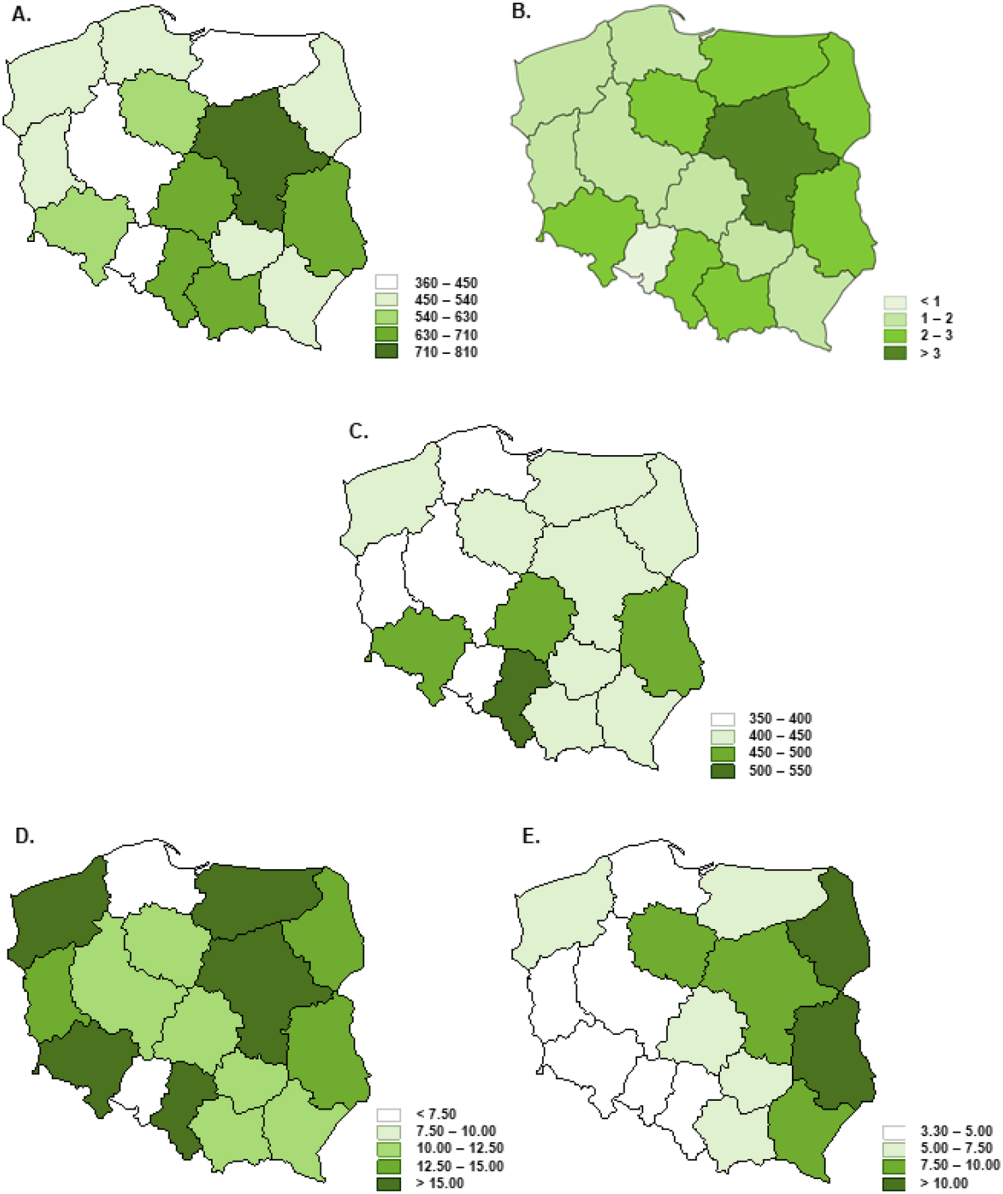
The spatial differentiation selected healthcare resources in Polish regions in 2019 where: **A**– The number of medical doctors per 100,000 citizens; **B**– The number of oncologists per 100,000 citizens; **C**– The number of hospital beds per 100,000; **D**– The number of hospital beds in the oncological departments per 100,000 citizens; **E**– The number of hospital beds in the infectious diseases departments per 100,000 citizens

Moreover, the situation is additionally complicated by national heterogeneity. The significant variation between regions concerns both: the density, wealth, and personal income structure as well as the healthcare resources. The differences in the selected socio-economic factors and healthcare resources between Polish regions [8] are presented in Figs. 1 and 2.

The clear differentiation of healthcare resources in combination with the longitudinal healthcare system’s underfunding has caused a system crash due to the COVID-19 pandemic, which has become the leading problem for public health worldwide.

**Fig. 3:**
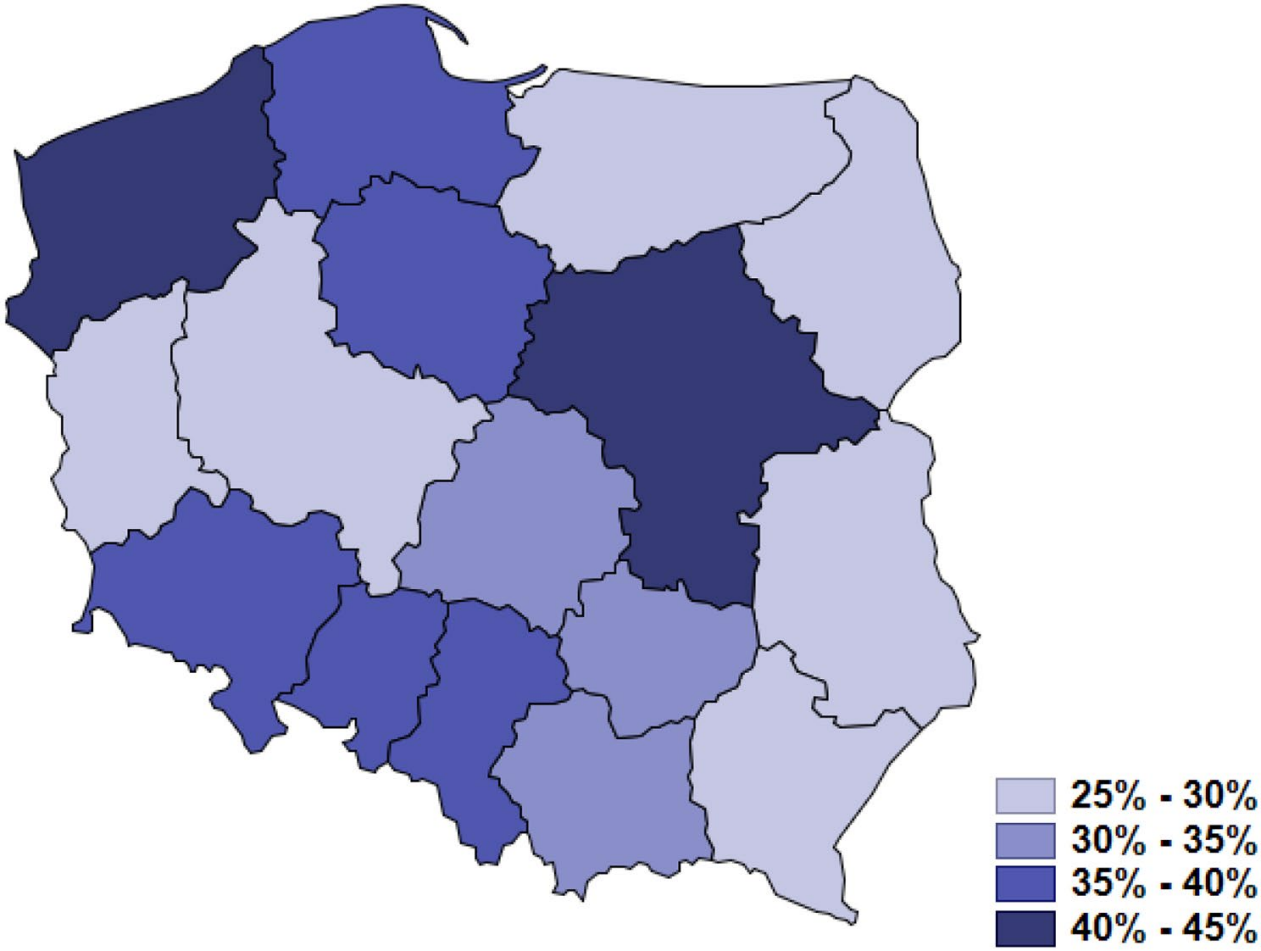
The percentage of teleconsultations of all granted services realized by the general practitioners in Polish voivodeships in 2020

For many years the delay in obtaining the specialized heathcare services and an aproprtiate oncological treatment after the diagnosis of cancer was a significant problem of the Polish healthcare system, that constituted a significant social and political issue. So that on January 1st, 2015 the Ministry of Health of Poland introduced the fast oncological diagnostics program and so-called “DILO cards” (Oncological Diagnosis and Treatment Card– in Polish: *Karta Diagnostyki i Leczenia Onkologicznego*), which were intended to ensure the fast access to the diagnosis and oncological treatment. If cancer is suspected based the patient receives the DILO card, which is registered in the IT system and it and provides access to a fast diagnostic and therapy. The DILO card is issued by the first medical doctor who suspects the cancer. These changes were aimed at reducing the delay in cancer diagnosis and treatment. Nevertheless, because of the pandemic restrictions and the authorities recommendations, many general practitioners and specialists have limited healthcare services to telemedicine only. Those could cause difficulties in diagnosing chronic diseases, such as cancers. Figure 3 presents the percentage of teleconsultations granted by the GPs in 2020 in relation to all settled services.

## Materials and methods

### Material

The analyses have focused on the most frequent cancers diagnosed in Poland in the 2015–2021 period, established based on the Polish National Cancer Registry 2021 report and our previous study concerning two Polish regions: the Silesian and Subcarpatian provinces [4]. It encloses malignant neoplasm of the colorectal– CRC (C18-C21 according to the ICD-10 classification), malignant neoplasm of bronchus and lung (C34), other malignant neoplasms of skin (C44), malignant neoplasm of kidney, except renal pelvis (C64) and malignant neoplasm of the breast (C50) among women or malignant neoplasm of the prostate (C61) in men.

In Poland, the National Cancer Registry (NCR) is collecting nationwide cancer data and publishing annual cancer bulletins since 1979 and it is one of the best cancer registries among EU member states [9]. The basis for editing the register is the cancer report form (MZ/N-1a) filled out by the physician who has diagnosed the cancer. Unfortunately, because of factors beyond the control of the authors of the NCR, such as organizational and economic issues that drastically affect cancer reporting, the NCR data and reports underestimate cancer incidence, as the authors themselves emphasize [4, 10]. So, additional data source is needed to assess more completely the cancer prevalence.

In our study, we have utilized data regarding patients aged over 20 years of life diagnosed with one of the above cancers who have obtained healthcare services in at least one of the over 250 specialized healthcare services provided to cancer patients such as nuclear medicine, brachytherapy, isotope therapy, services of the clinical oncology, chemotherapy, oncological surgery and services, cancer drug programs, comprehensive products dedicated for the colorectal and breast cancer patients and diagnostic radiology settled by the National Health Fund of Poland. Those patients have obtained services in the following ranges: (1) hospitalization in the clinical oncology units (including one-day hospitalizations), (2) hospitalizations in the oncological radiotherapy units (including one-day hospitalizations and all organizational forms of radiotherapy services), (3) hospitalizations in oncological and general surgery units and (4) outpatient oncological specialist healthcare.

The children as well as the young adults (aged under 20) were not included in the study. Finally, we have analysed the data concerning 803,146 patients of which 166,018 were colorectal cancer cases, 147,299– were lung and bronchus cancer, 122,549– had skin cancer, 56,150– had kidney cancer and 179,564 women suffering from breast cancer and 131,566 of men with the prostate cancer.

The pre-processed, secondary epidemiological data concerning the incidence of each of the analysed cancers in subsequent years were obtained from the National Health Fund of Poland (NFZ) which is the only organization responsible for the contracting and accounting of healthcare services financed from public funds in Poland. So, the NFZ may be perceived as the most reliable and comprehensive data source.

### Methodology

**Fig. 4:**
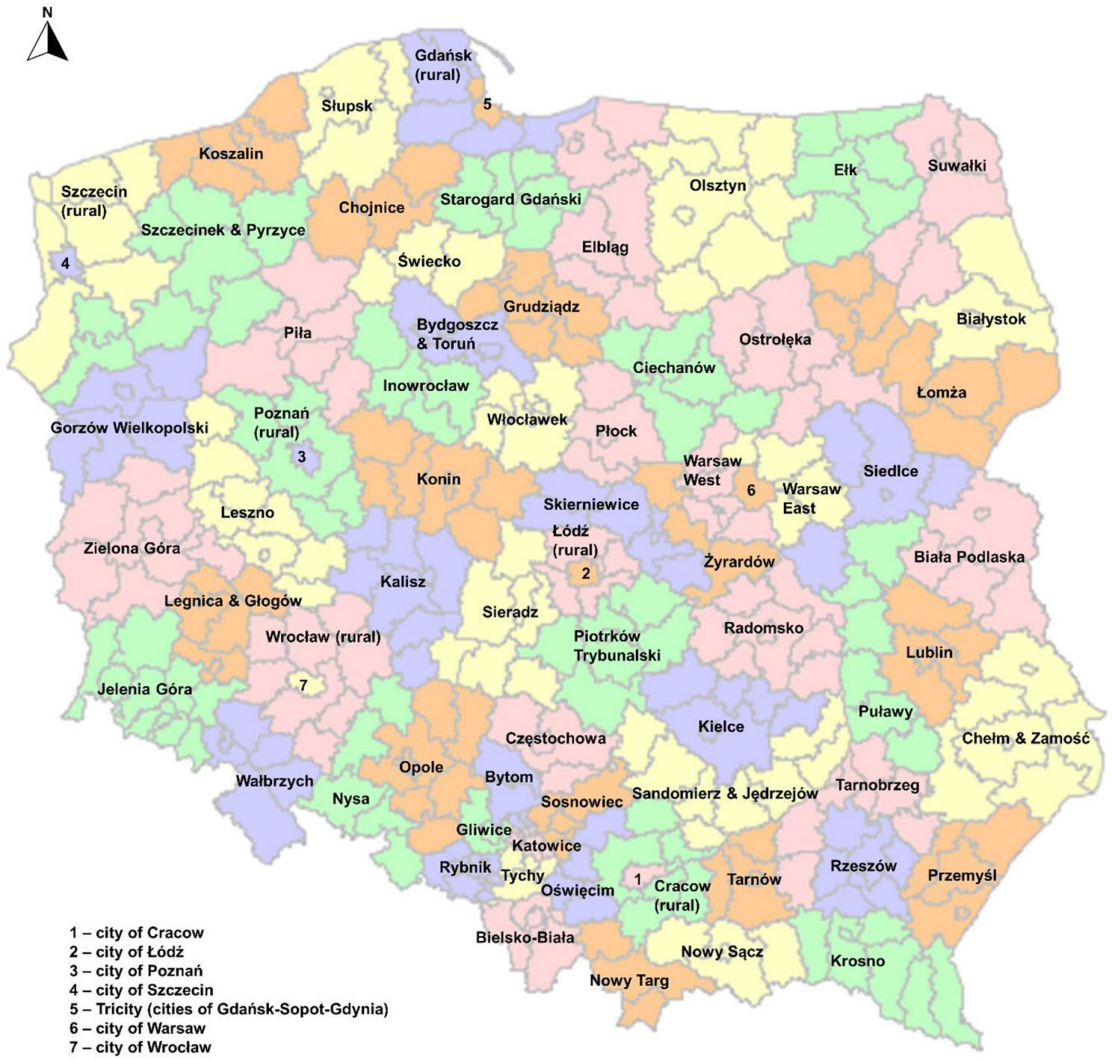
The division of Poland into subregions (NUTS-3 units)

The epidemiological analysis was performed and divided into subregions (NUTS-3 units), according to patients’ domicile at the time of cancer diagnosis. The NUTS classification was formally introduced in Poland on November 26th, 2005 as the consequence of Poland’s accession to the European Union (on May 1st, 2004). As a result of the subsequent revisions of the NUTS classification, 73 subregions have been established. Figure 4 presents the map of Poland with marked subregions, additionally, the lines show the borders of the counties.

The time points of providing the first healthcare service were considered as the time of cancer diagnosis.

Unlike the Polish National Cancer Registry which bases its annual rapports on the MZ/N-1a form filled in by the physician who has diagnosed cancer, we have based on the data from the medical treatment billing process by the NFZ.

We had chosen the years 2015, 2019, and 2020 as the three time points for which the standardized incidence rates (SIR) were calculated to assess the basic level of cancer morbidity as well as the effect of the COVID-19 pandemic.

For each of the 73 Polish subregions, we have calculated the incidence rate per 100,000 population standardized to the European Standard Population 2013 (ESP 2013), with the use of the formula:

$$SIR = {{\sum\limits_{i = 1}^N {{{{k_i}} \over {{n_i}}} \cdot {w_i}} } \over {\sum\limits_{i = 1}^N {{w_i}} }}$$

where:

*N*– number of five-year age groups,

*k*_*i*_– number of cases in an *i*-five-year age group,

*n*_*i*_– population size in an *i*-five-year age group,

*w*_*i*_– weight assigned to an *i*-five-year age group based on ESP 2013.

To assess the annual change of SIR for each cancer that has been analyzed, the Annual Percent Change (APC) in the subregions was determined with the use of the formula:

$$ APC=100\cdot \left({e}^{b}-1\right)$$

where:

*e*– Euler’s number ($$ \cong $$2.718),

*b*– linear regression coefficient for the ln(rates) ∼ calendar year model.

The assessment of the statistical significance of APC was equivalent to the statistical evaluation of the linear regression model. Statistical significance was set at a *p*-value below 0.05. Two-tailed tests were used.

The statistical analyses as well as the mapping of SIR values were performed with the use of the *Statistica* v. 13.3 software (TIBCO Software Inc., Palo Alto, CA, USA).

Data on the state of population in each of the subregions as well as selected social and economic factors were obtained from the Statistics Poland database.

### Ethical approval

According to the Bioethical Committee of the Medical University of Silesia (No PCN/CBN/0052/KB/108/22), this study did not require the permission of the Committee due to its retrospective character.

All methods were conducted following relevant guidelines and regulations and respecting the confidentiality of biomedical data.

## Results

The nationwide pre-processed data concerning cancer cases shared by the NFZ have been analyzed. In the 2015–2019 period, before the COVID-19 pandemic occurred, the nationwide incidence of most of the analyzed cancers remained stable or slightly decreased (as the lung cancer) but the prevalence of the prostate cancer has increased. Simultaneously, the large differences between subregions with the least and the highest incidence were observed, what shows the Table 1.

**Table 1:** The nationwide average SIR with the lowest and highest values across the country

		C18 - C21	C34	C44	C64	C50	C61
**2015**	**National Average**	**90.4**	**83.0**	**68.8**	**32.7**	**164.0**	**151.8**
	Min	60.6	50.0	22.7	20.3	104.4	96.1
	Max	149.1	216.1	139.9	64.5	267.9	441.0
**2016**	**National Average**	**88.1**	**79.4**	**68.5**	**31.6**	**160.8**	**166.2**
	Min	56.3	52.2	19.1	15.1	107.0	99.8
	Max	150.8	191.9	157.2	60.7	249.2	459.4
**2017**	**National Average**	**86.8**	**75.8**	**67.1**	**28.8**	**160.1**	**169.0**
	Min	60.7	46.6	25.8	15.1	106.3	107.2
	Max	155.9	145.8	142.0	58.3	251.4	384.3
**2018**	**National Average**	**88.2**	**73.1**	**68.2**	**27.6**	**161.0**	**163.3**
	Min	55.5	48.1	24.3	17.5	113.4	102.6
	Max	166.7	146.9	132.5	54.5	251.7	279.3
**2019**	**National Average**	**88.8**	**72.4**	**69.8**	**28.4**	**165.9**	**173.5**
	Min	61.8	44.4	29.1	16.6	111.1	109.2
	Max	145.0	152.1	133.8	53.8	263.4	336.9
**2020**	**National Average**	**76.2**	**63.2**	**50.2**	**23.3**	**147.5**	**143.4**
	Min	58.9	41.5	18.4	13.8	95.9	97.2
	Max	140.4	136.2	93.2	40.2	254.3	238.7
**2021**	**National Average**	**84.0**	**67.0**	**64.4**	**25.5**	**168.2**	**167.2**
	Min	54.1	45.3	24.4	15.7	110.2	98.5
	Max	143.4	133.0	114.9	47.0	270.9	288.8

The further figures regarding each cancer show the SIR values in each of the subregions (additionally, the lines show the borders of counties) in 2015, 2019, and 2020. The annual percentage changes nationwide as well as in the each of subregions in the 2016–2021 period are presented below on the charts and maps, respectively.

Additionally, the APC calculated for all the analyzed cancers in each of the subregions is presented in the Supplementary Table.

**Fig. 5:**
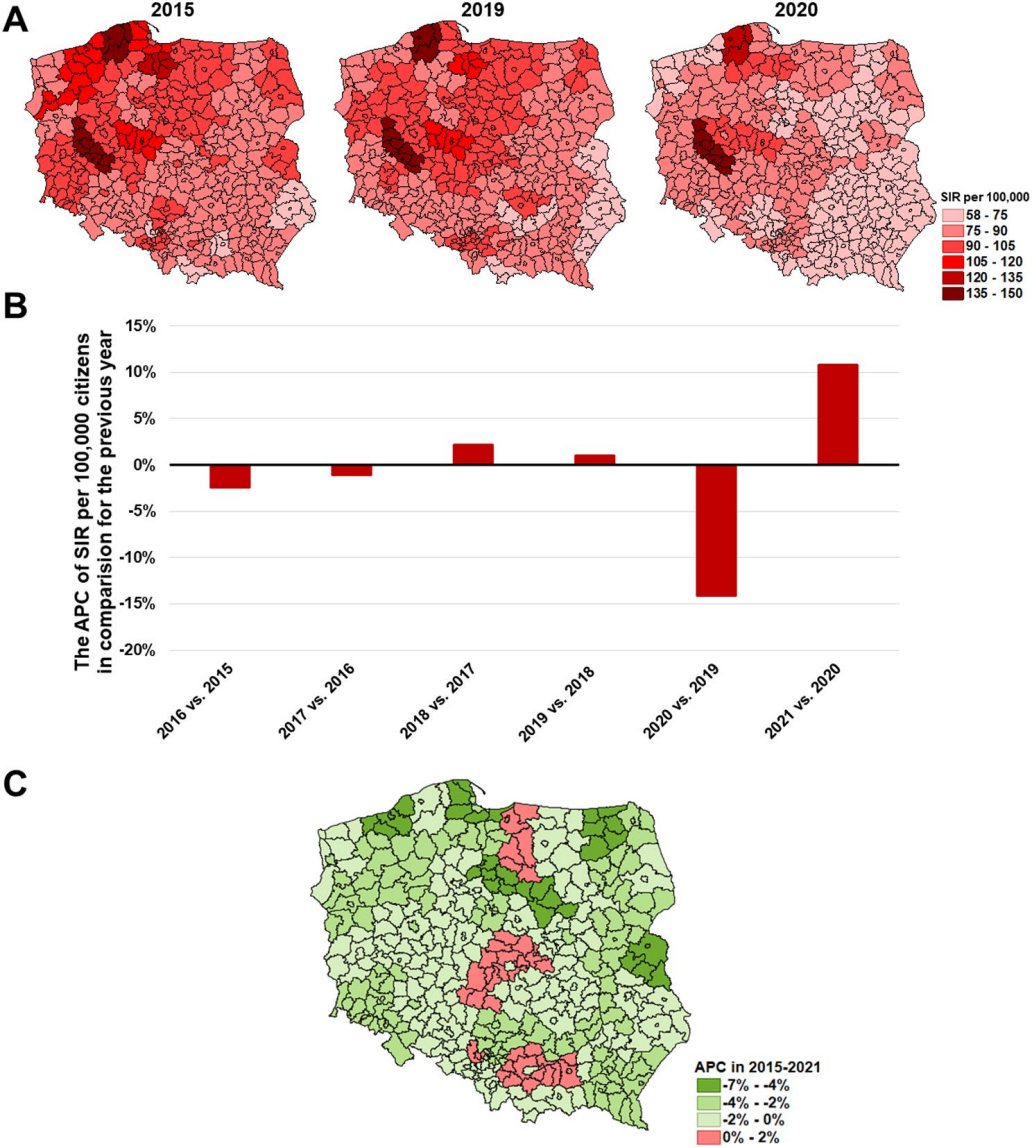
The epidemic situation of colorectal cancer **(C18-C21)** in Poland where: **A**– SIR values in 2015, 2019, and 2020 in each of the subregions; **B**– the nationwide APC of SIR in subsequent years; **C**– the APC in 2015–2021 period in each of the subregions

Colorectal cancer (C18-21) has become the leading oncological problem in Poland in recent years. In 2015 the greatest incidence of C18-21 was observed in the Leszno, Słupsk, and Starogard subregions (149.1, 138.8 and 120.5 per 100,000 respectively), and in the 2015–2019 period, unstigmatized by the pandemic, the SIR remained stable (Fig. 5A). Subsequently, in 2020 a significant decrease in the SIR was observed in most of the subregions beside the Leszno and Słupsk in which SIR values were 145.0 and 143.1 per 100,000 respectively, with clear spatial division between northern-west and southern-east areas. The long-term trend shows a significant increase in the SIR in 2021 compared to 2020 (Fig. 5B). The only subregions where the Annual Percent Change (APC) has increased despite the pandemic effect were: Elbląg, Gliwice, Cracow (rural), Łódź, Tarnów, and Skierniewice, although APC changes were not significant (Fig. 5C and the Supplementary Table).

**Fig. 6:**
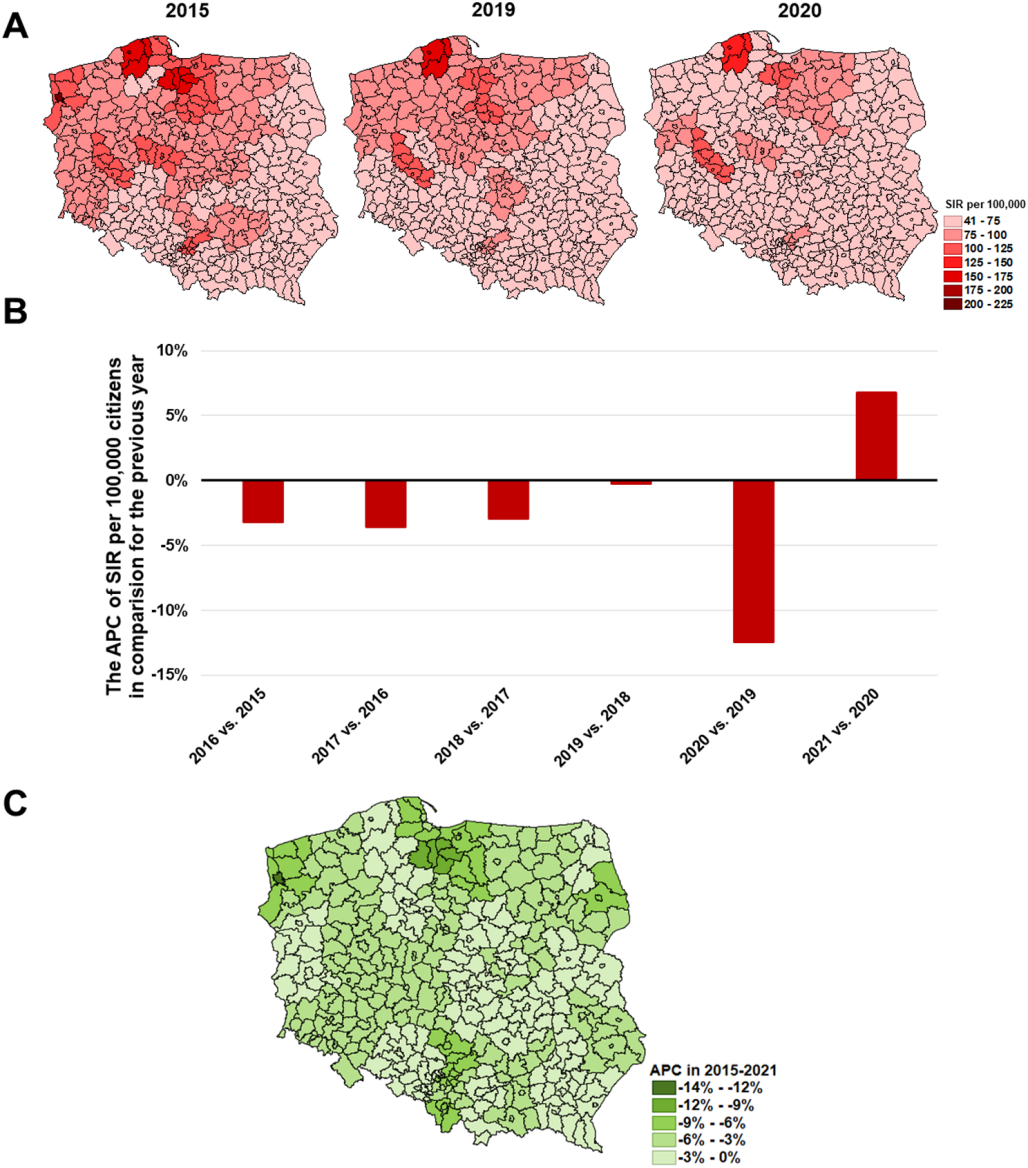
The epidemic situation of bronchus and lung cancer **(C34)** in Poland where: **A**– SIR values in 2015, 2019, and 2020 in each of the subregions; **B**– the nationwide APC of SIR in subsequent years; **C**– the APC in 2015–2021 period in each of the subregions

The greatest SIR of lung cancer (C34) in 2015 was observed in the city of Szczecin as well as Słupsk and Starogard subregions (216.1, 172.1, and 167.0 per 100,000 properly). Then, in 2015–2019 the SIR decreased across the country (in most of the subregions statistically significant) remaining the greatest prevalence in the Słupsk, Starogard Gdański and Leszno subregions (152.1, 112.6, and 105.9 per 100,000 correspondingly) (Fig. 6A). In 2020 compared to 2019, the SIR decreased by -12.8% nationwide, but it increased by 6.5% in 2021 (Fig. 6B). The greatest reduction of C34 incidence was observed in the city of Szczecin with the APC − 13.99 (*p* < 0.05) (Supplementary Table).

**Fig. 7:**
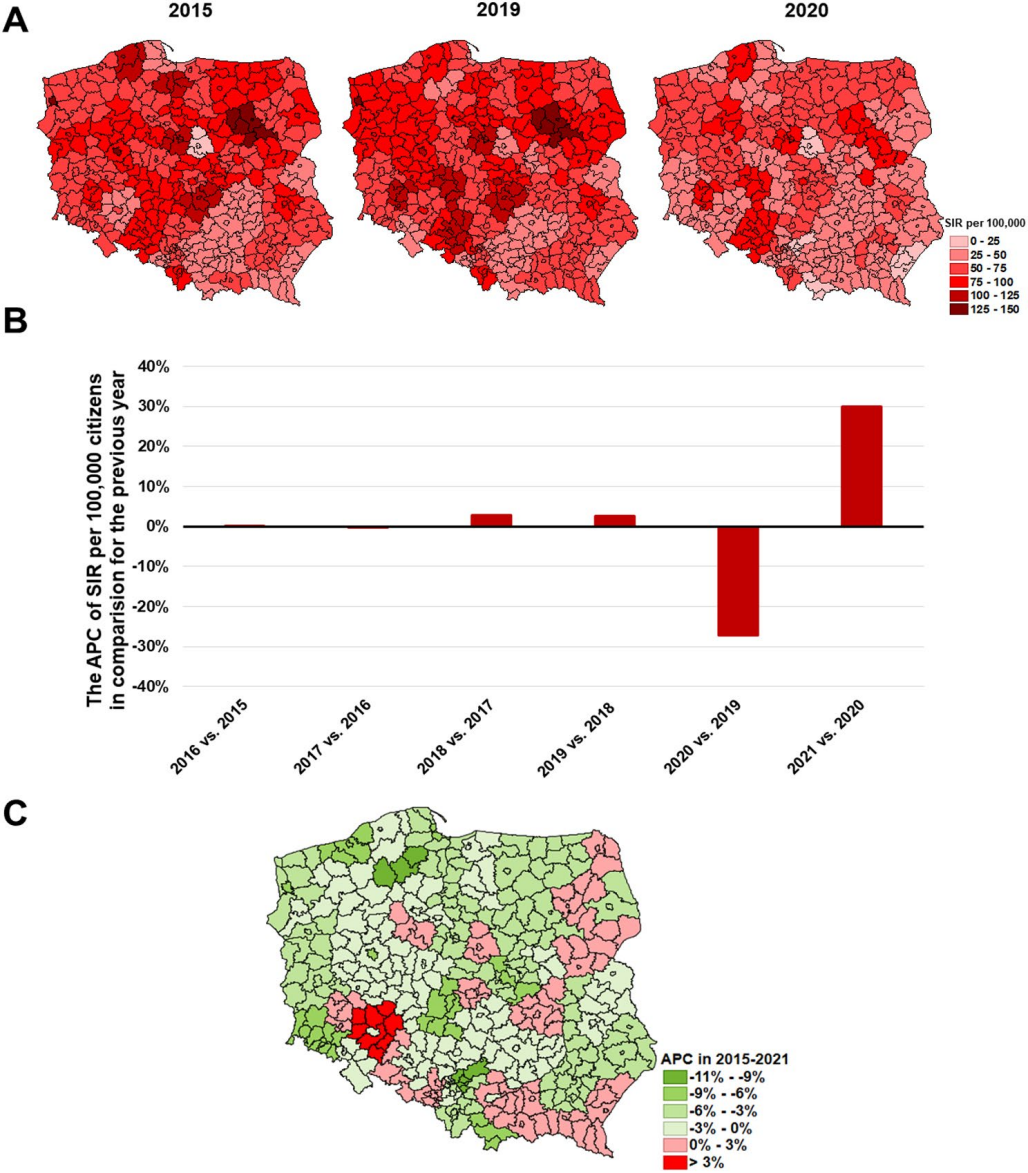
The epidemic situation of skin cancer **(C44)** in Poland where: **A**– SIR values in 2015, 2019, and 2020 in each of the subregions; **B**– the nationwide APC of SIR in subsequent years; **C**– the APC in 2015–2021 period in each of the subregions

The prevalance of skin cancer (C44) in 2015 was comparable across the country. Nevertheless, the greatest SIR was observed in the cities of Poznań and Szczecin, Ostrołęka, and Włocławek subregions (139.9, 128.9, 127.1 and 116.8 per 100,000 respectively) and it did not change till 2019 (Fig. 7A). Subsequently, just like with the lung cancer, the SIR decreased by -27.2% in 2020 and then increased by 30.0% in 2021 (Fig. 7B).

The APC changes show that the C44 burden slightly decreased in 2015–2021 in most of the subregions (Fig. 7C). The Wrocław (rural) subregion has stood out as the one with a greater increase of the C44 incidence (41.6 per 100,000 in 2015 and 58.2 in 2021).

**Fig. 8:**
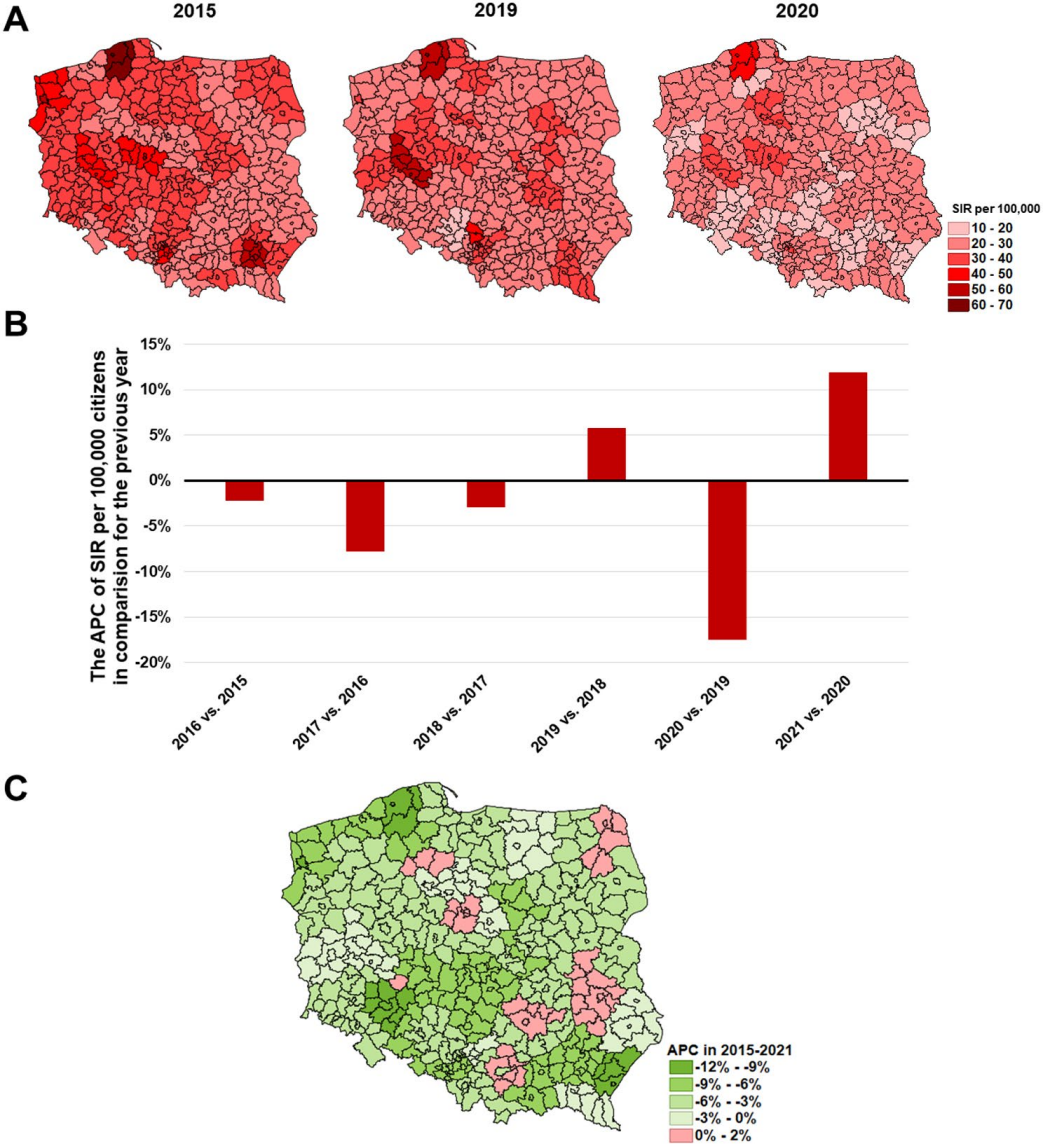
The epidemic situation of kidney cancer, except the renal pelvis **(C64)** in Poland where: **A**– SIR values in 2015, 2019, and 2020 in each of the subregions; **B**– the nationwide APC of SIR in subsequent years; **C**– the APC in 2015–2021 period in each of the subregions

In 2015–2021 the burden of kidney cancer (C64) decreased in most of the Polish subregions (Fig. 8) and the most noticeable reduction was observed in the Przemyśl, Słupsk, and Wrocław (both city and rural) subregions (the APC − 9.03, -9.28, -11.20 and − 9.15 respectively) (Supplementary Table).

**Fig. 9:**
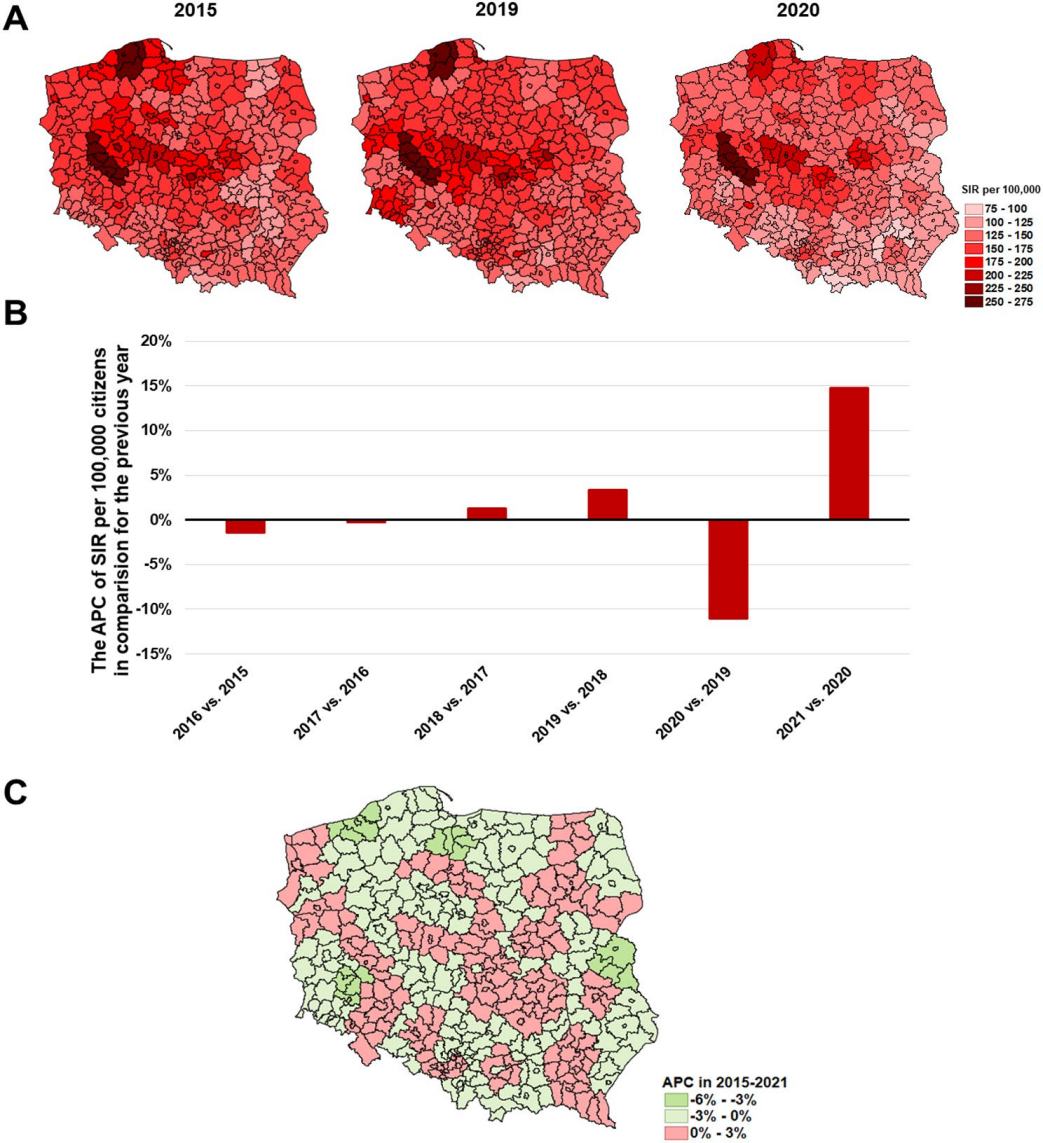
The epidemic situation of breast cancer **(C50)** among women in Poland where: **A**– SIR values in 2015, 2019, and 2020 in each of the subregions; **B**– the nationwide APC of SIR in subsequent years; **C**– the APC in 2015–2021 period in each of the subregions

Similarly, to most developed societies in Western Europe, breast (C50) and prostate (C61) cancers were the most frequent cancers diagnosed in Poland among women and men respectively. In 2015 the greatest SIR for C50 was found in the subregions of Słupsk (267.9 per 100.000), Leszno (266.0), cities of Łódź, Poznań and Warsaw (238.8, 232.7 and 221.7 properly) and subregions of Tricity (219.9) and Konin (207.1). Such a situation has remained stable till 2020 when the COVID-19 pandemic has occurred. In 2020, compared to 2019 a visible reduction of SIR in most of the subregions was found, especially in the eastern areas (e.g. Tarnobrzeg subregion with SIR 126.8 in 2019 and 95.8 in 2020). The least impact on the C50 SIR, the pandemic has had in the Konin, Leszno, Słupsk subregions, and the city of Warsaw (Fig. 9).

**Fig. 10:**
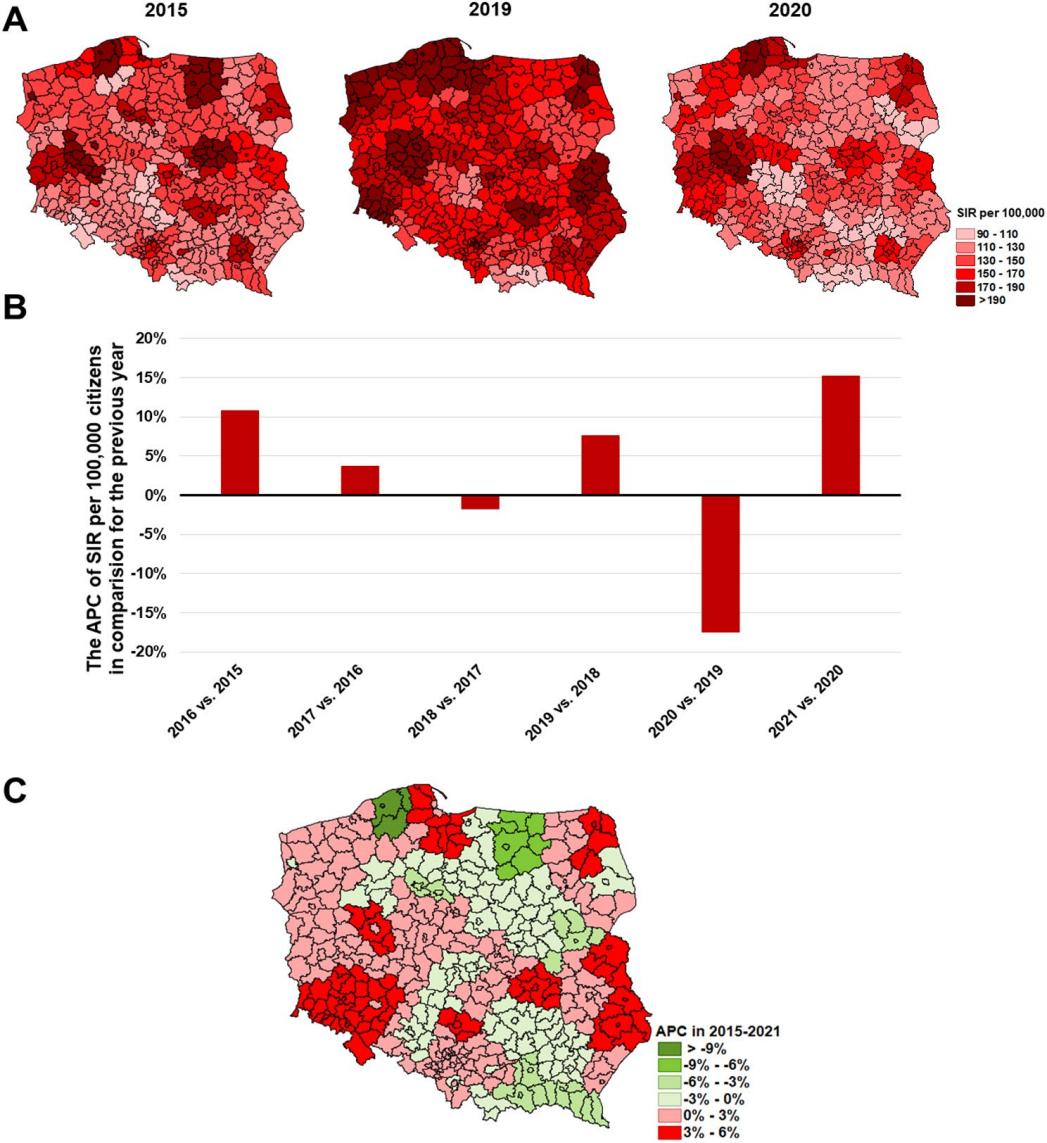
The epidemic situation of prostate cancer **(C61)** in Poland where: **A**– SIR values in 2015, 2019, and 2020 in each of the subregions; **B**– the nationwide APC of SIR in subsequent years; **C**– the APC in 2015–2021 period in each of the subregions

The prevalence of prostate cancer increased in the 2015–2019 period in most of the Polish subregions (Fig. 10A). Nevertheless, besides the increase of prostate cancer morbidity at the national level, in some subregions we have found a reduction of this burden; the greatest decrease of APC was found in the subregions of Słupsk (-9.22) and Olsztyn (-6.24). Of note, the Słupsk subregion was also one of the subregions with the greatest incidence rate of prostate cancer in 2019 (336.9 per 100,000). The greatest SIR values, over 190 per 100,000 were found in the subregions of Biała Podlaska, Chojnice Gdańsk, Jelenia Góra, Kielce, Koszalin, Leszno, Lublin Poznań (both city and rural), Szczecin (both city and rural), Starogard, Suwałki, and Tricity (Supplementary Table).

In 2020 when the COVID-19 pandemic occurred, a visible reduction of the SIR of C61 in most of the subregions was found.

## Discussion

In the Polish healthcare system, the National Health Fund of Poland is the only institution contracting and accounting the healthcare services financed by public funds. Citizens paying mandatory health insurance are entitled to healthcare access. Simultaneously, the private health insurance sector is poorly developed so the NFZ nationwide data can be considered as the most complete and reliable. So, our study has covered the Polish population.

### The cancers epidemiology

In the current study, we have analyzed selected the most common cancers diagnosed in Poland in 2015–2021, based on the National Cancer Registry database and our previous study concerning two regions of southern Poland: the Silesian and Subcarpathian voivodeships [5]. It was colorectal cancer, lung and bronchus cancer, skin cancers (without melanoma), kidney cancer except for renal pelvis, and two gender-related cancers: breast cancer among women and prostate cancer among men. Such structure of most prevalent cancers does not differ from Western societies with high Human Development Index (HDI). Our results correspond with available data [9].

One of the most common cancers diagnosed in Poland in 2015–2021 was colorectal cancer. This disease remains the third of the most frequent cancers diagnosed worldwide and the second cause of cancer-related deaths because of insufficient diagnostics and ineffective therapy [2, 11–13]. The high incidence of colorectal cancer observed in Poland might be affected by the socio-economic transformation of the 1990s and progressive changes in lifestyle and Western diet change as well as the low utilization of screening programs (such as screening colonoscopy). The high prevalence of low physical activity, overweight and obesity, smoking, and excessive alcohol consumption remain serious problems for the nationwide health policy. The stabilization of the colorectal SIR in 2015–2019, observed in our study, corresponds with the Xi and Xu findings, according to which in the high-developed countries the burden of CRC stabilizes at a relatively high level, over four times greater compared to the countries of low HDI [2].

The lung cancer were the second of the most frequent cancers diagnosed nationwide in 2015–2021 without gender differences. The available data underline the relationship between smoking and air pollution and the risk of lung cancer [14–18]. Also, in Poland lung cancer incidences and air pollution are linked [19], especially that according to the World Bank Group, Polish cities remain 36 of 50 of the most polluted urban areas of the European Union [20].

Our results show that the incidence rate of lung cancer has decreased in 2015–2021 all over the country. This could be affected by the social changes already observed over ten years ago resulting from the decrease in the smoking prevalence in 2015 compared to 1999 [21] and the changes in the smoker’s structure [3].

The high prevalence of skin cancer (C44) observed in our study does not deviate from most of the countries with the domination of Caucasians. In the last thirty years, skin cancer morbidity has tripled in the United States and Europe [22].

The decrease in skin cancer’s APC in most of the northern and northern-west subregions may be associated with the limitations of the healthcare system resources such as low numbers of healthcare providers and available assets, such as in-hospital beds and the limited number of medical doctors in those areas (Fig. 1a and c). Such findings correspond with Augustin et al. results indicating that the availability of medical doctors, especially general practitioners, and potential difficulties in accessing them are associated with the number of skin screening tests [22].

Breast and prostate cancers were the most frequent oncological diseases diagnosed in Poland in 2015–2021 among women and men, respectively. Those cancers are the most frequent oncological diseases diagnosed in highly developed countries.

In our study we found increased morbidity for prostate cancer in 2015–2019, meanwhile, the prevalence of breast cancer remained stable. The increased burden of breast cancer in 2015 was observed in wealthy subregions including the largest urban centers: cities of Łódź, Poznań and Warsaw as well as the subregions of Tricity and Konin. Those findings correspond with previous results. Pacelli et al. have shown that after the period of increase of incidence the morbidity and mortality of breast cancer in most developed countries have stabilized or even decreased slightly in the last 15–20 years [23]. It may be caused by the wide spreading of social campaigns and screening programs reducing inequalities and improving accessibility of mammography [24]. Moreover, when assessing the breast cancer burden, especially in survivors, we should not ignore the progress in the therapy, in particular HER2-positive and luminal subtypes. The increased morbidity of breast cancer, observed in our study, corresponds with Didkowska et al. results [9].

We found an increased SIR of prostate cancer in 2015–2019 as well. According to the previous study, the C61 APC was the highest among the most common cancers diagnosed in Poland [9]. The etiology of prostate cancer is relatively weakly examined compared to other cancers. The early diagnosis is essential but over 75% of men aged over 45 have never performed the PSA measurement.

### The COVID-19 impact

In 2020 a visible decrease in the SIR for each of the analyzed cancers was observed. It is most likely related to the limited accessibility and availability of healthcare services for non-COVID diseases because of healthcare staff and resources’ engagement in fighting the COVID-19 pandemic. The second important aspect was the procrastination of not requiring urgent medical consultations, physical examinations, and diagnostic procedures [25]. This was caused both by the care of the medical professionals’ maintenance and patients’ and physicians’ fear of COVID-19 infection, especially before the vaccine development.

The COVID-19 pandemic has exposed health inequalities and had a dramatic impact on cancer patients. Due to pandemic restrictions, most healthcare services have been realized with the use of remote methods, such as telemedicine. The traditional face-to-face medical consultations with physical examination had been limited [26, 27]. The percentage of physicians’ teleconsultations increased from 1.2% in March to over 60% in April 2020 [28]. Such patients’ management in the pandemic reality was difficult and probably in many cases delayed diagnosis, and treatment, and have affected the suboptimal therapy. Moreover, cancer patients had a higher risk of SARS-CoV2 infection and complications [25, 28].

Our results show a print of such unfavorable pandemic conditions by the visible reduction of SIR of each of the analyzed cancers in 2020.

In 2019, just before the pandemic occurred, the burden of colorectal and lung cancer had been characterized by a clear spatially dependent decreasing division along the northern-west– southern-east axis and these findings correspond with the previous results [9]. Subsequently, in 2020 the incidence of colorectal cancer has decreased especially in the eastern subregions (those areas are characterized by relatively unfavourable economic conditions) while the lung cancer incidence rate has decreased equally across the country. The reduction of colorectal cancer was most likely affected by the abandonment of standard medical consultations and the replacement of them by remote methods. It caused a drastic limitation in the number of performed endoscopic [27].

In 2020, compared to the period preceding the COVID-19 pandemic, the slightest decrease in the incidence of CRC was in subregions of Gliwice, Łódź, Tarnów, and Cracow– rural. Those areas are large urban centers with well-developed healthcare infrastructure, including diagnostic and oncological resources. Despite the involvement of the healthcare system in the fight against the pandemic, patients living in these areas were in a more favorable situation enabling the treatment and diagnosis of diseases other than COVID-19.

The visible SIR decreasing in the northern-west– southern-east axis in 2020 was observed in the case of skin cancer and breast cancer among women as well. The subregions of southern-east areas of Poland are mainly rural with relatively poor and unevenly allocated healthcare resources, especially specialized ones, e.g. lower number of the hospital beds in the oncological departments per 100,000 citizens and medical doctors combined with unfavorable economic status below the national average (as presented in Figs. 1 and 2). The pandemic-related limited access to healthcare supplies in rural areas may be the consequence of the insufficiency of the healthcare system and human resources [26]. However, it cannot be ignored that this problem is multi-faceted and it depends on the combined impact of many factors such as socio-economic, environmental, and the availability and organization of healthcare and it requires further analyzes of the impact of each factor at the local level.

The spatial differentiation of the cancer prevalence as well as the health inequalities were observed in many countries, including European states. The social determinants of health inequalities are well established but there is no exhaustive data on the spatial ones. In France at the beginning of the 21st century, the standardized mortality rate of cancers in the northern counties was almost twice as high as in the southern areas, which was caused by environmental, social, and spatial conditions. The spatial differentiation of the cancer burden was affected by the unequal access to healthcare resources occurring by the smaller use of medical services in the areas with a smaller number of medical entities. This contributes to less favorable prevention, early diagnostics, and appropriate patient management. So that remote patients have worse access to specialized health centers and lower survival chances [29].

The spatial differentiation of the cancer prevalence was found in Slovakia as well. Slovakia is close to Poland because of the similarities in the social and political transformation observed at the turn of the 80s and 90s. Since the middle of the 80s, the burden of communicable diseases had decreased, and cancers became the leading health problem in Slovakia. Moreover, the significant disproportion between the wealthy regions and the southern and western areas is noticeable both as the inequality of the socio-economic factors as well as the better quality of life and healthcare [30].

The Polish healthcare system has been underfinanced for many years and successive governments since 1990 it has been occurring as one of the leading problems that has to be solved.

In 2005 and 2019 Poland spent 1.05 and 2.3 billion euro on cancer treatment respectively [31]. The increase in the finances dedicated to oncology was associated with the development and implementation of the so-called “oncological package” which was supposed to increase the availability and efficiency of oncological healthcare. Simultaneously, according to the National Cancer Strategy 2020, one of the milestones was to increase the percentages of 5-year survivals in cancer diseases and improve the prevention and screenings [9]. According to the EUROCARE-5 study, the cancer 5-year survival rate noted in Poland was significantly lower than the EU average (43% vs. 54.6%). Those targets were essential, from the public health’s point of view because according to WHO, over 40% of cancer incidences might be avoided or successfully treated in case of early detection [32].

Unfortunately, the cancer prevention expenditures in 2012–2015 decreased by 10% [33] as a low percentage of Poles utilized screening tests, such as colonoscopy or mammography, despite the access to these programs.

## Limitations

Our study has some limitations resulting from the principles of organization and settlement of health services. Between the diagnosis of cancer by the medical doctor during examination and the patient’s referral for further diagnostics services and oncological therapy granted in one of the scopes included in our analyses there is a “time window” causing a slight incidence ratios shift in time. Nevertheless, we trust that it does not strongly affect the comprehensiveness and reliability of our results.

## Conclusions

The burden of cancer in Poland is spatially differentiated and the time changes of each of the analysed cancers differ. While the prevalence of colorectal, skin, and breast cancers remained stable, the incidence of lung bronchus and kidney cancers decreased but the incidence of prostate cancer increased at the same time.

Subsequently, a significant decrease in the SIR of the most frequent cancers diagnosed in Poland in 2020 compared to 2019 was observed and afterward, a significant increase in 2021 was noted, most likely due to the gradual reduction of epidemic restrictions. It should be assumed that many patients whose cancer diagnosis and beginning of treatment were delayed, because of pandemic restrictions and inaccessibility of healthcare services, could be diagnosed with a more advanced stage. Those implicate a worse prognosis and diminished chance for cure.

### Electronic Supplementary Material

Below is the link to the electronic supplementary material.

Supplementary Material 1

## Data Availability

Secondary epidemiological, depersonalized data was obtained from the National Health Fund of Poland (NFZ) after the healthcare services settlement process. Disclosure of data requires the consent of the National Health Fund of Poland, as data administrator. The datasets analysed during the current study are not publicly available but it is possible to obtain them through access to public information. For this purpose, a written request addressed to the Director of the National Health Fund of Poland should be made.
