# Supplementary material for: The epidemiology of the most frequent cancers in Poland in 2015–2021 and the impact of the COVID-19 pandemic on cancer incidence
Source: Arch Public Health. 2024 Apr 15;82:49. doi: 10.1186/s13690-024-01277-6 (PMC11021004; doi:10.1186/s13690-024-01277-6)
Supplement: Supplementary file 1 — Supplementary Material 1 [file 13690_2024_1277_MOESM1_ESM.docx]

**Supplement**

|  | **The Annual Percent Change** | | | | | |
| --- | --- | --- | --- | --- | --- | --- |
| **Subregion** | **C18-C21** | **C34** | **C44** | **C67** | **C50** | **C61** |
| BIAŁA PODLASKA | -5,33** | -1,73 | -2,52 | -4,76 | -5,04*** | 5,88 |
| BIAŁYSTOK | -0,52 | -7,11* | -3,33 | -3,18 | -0,59 | -1,39 |
| BIELSKO - BIAŁA | -0,17 | -6,05* | -4,90 | -5,60* | -1,17 | 0,00 |
| BYDGOSZCZ & TORUŃ | -1,60 | -2,14 | -0,34 | -1,17 | -1,86 | -4,08 |
| BYTOM | -3,25 | -2,25 | -2,97 | -4,25 | -0,27 | 2,33 |
| CHEŁM & ZAMOŚĆ | -1,61 | -3,42** | -3,96* | -0,53 | -0,25 | 3,34 |
| CHOJNICE | -2,27 | -0,69 | -9,44* | -6,33 | -2,34 | 1,72 |
| CIECHANÓW | -5,01* | -2,23 | -5,90** | -7,22* | -1,79 | -2,57 |
| CZĘSTOCHOWA | -3,75* | -6,24** | -1,41 | -7,58** | -1,86 | 5,57 |
| ELBLĄG | 0,27 | -6,42*** | -2,58 | -4,25** | -0,38 | -0,38 |
| EŁK | -4,13 | -5,45* | -4,57* | -3,07 | 1,31 | 1,97 |
| GDAŃSK | -4,19 | -8,61** | -3,94 | -3,69* | -1,91 | 3,02 |
| GLIWICE | 1,05 | -1,56 | 0,51 | -3,96 | -1,81 | 2,20* |
| GORZÓW WIELKOPOLSKI | -2,57 | -1,57* | -5,84 | -5,97 | 0,50 | 2,06 |
| GRUDZIĄDZ | -5.00* | -3,53 | -4,34 | -2,33 | 0,71 | -2,15 |
| INOWROCŁAW | -1,63 | -2,56 | 0,20 | -3,51 | -1,67 | 0,04 |
| JELENIA GÓRA | -2,19 | -4,32* | -7,05 | -4,90 | -2,02 | 3,98 |
| KALISZ | -0,89 | -3,64* | -0,99 | -7,28** | -0,02 | 1,57 |
| KATOWICE | -2,25* | -8,00** | -5,23 | -8,34*** | 1,12 | 1,63 |
| KIELCE | -1.00 | -2,74 | -0,18 | 0,71 | 0,20 | -2,36 |
| KONIN | -0,71 | -5,29** | -1,16 | -5,45** | 0,27 | 2.00 |
| KOSZALIN | -6,61** | -5,13* | -6,31* | -6,73* | -3,15 | 2,86 |
| CRACOW (RURAL) | 0,23 | -2,08 | 0,06 | 1,24 | 0,43 | 0,22 |
| CITY OF CRACOW | -0,93 | -2,18 | -0,16 | -3,52 | -0,35 | -0,04 |
| KROSNO | -2,41 | -1,99 | 0,29 | -2,68 | 0,39 | -5,08 |
| LEGNICA & GŁOGÓW | -3,73** | -5,76*** | 0,78 | -5,26 | -4,28 | 4,20* |
| LESZNO | -0,44 | -3,64 | -2,09 | -0,60 | 0,51 | 1,59 |
| LUBLIN | -1,19 | -3,48 | -1,81 | 1,64 | 0,59 | 1,21 |
| ŁOMŻA | -2,69 | -5,17 | 0,77 | -5,41* | 0,38 | 0,04 |
| ŁÓDŹ (RURAL) | 1,03 | -1,39 | 0,01 | -6,12** | 1,42 | -0,31 |
| CITY OF ŁÓDŹ | -1,83 | -1,99 | 0,02 | -6,67* | -2,15 | -1,33 |
| NOWY SĄCZ | -0,92 | -4,13* | 0,02 | -6,17* | -0,53 | -4,41 |

*** - p < 0.05 ** - p < 0.01 *** - p < 0.001**

| **Subregion** | **C18-C21** | **C34** | **C44** | **C64** | **C50** | **C61** |
| --- | --- | --- | --- | --- | --- | --- |
| NOWY TARG | -0,29 | -1,82 | -8,37 | -5,28 | -0,46 | -2,66 |
| NYSA | -0,36 | -2,45 | 1,17 | -6,32 | -2,80 | 0,33 |
| OLSZTYN | -1,16 | -4,02** | -4,67 | -1,57 | -0,35 | -6,24** |
| OPOLE | -1,28 | -1,75 | -1,09 | -4,96 | 0,38 | -1,07 |
| OSTROŁĘKA | -0,28 | -3,27 | -4,01 | -3,14 | 2,15 | -2,21 |
| OŚWIĘCIM | 1,53 | -3,69* | -5,16 | -3,05* | -0,20 | 0,35 |
| PIŁA | -2,66 | -3,15 | -2,61 | -3,05 | -1,10 | -1,31 |
| PIOTRKÓW TRYBUNALSKI | -1,20 | -2,55 | -0,69 | -6,97* | 1,10 | 1,43 |
| PŁOCK | -1,32 | -1,63 | 0,86 | -0,53 | 0,01 | -1,09 |
| CITY OF POZNAŃ | -2,46 | -4,92* | -6,43* | -4,38* | -1,16 | 1,66 |
| POZNAŃ (RURAL) | -0,55 | -4,98* | -0,55 | -4,04 | -0,58 | 3,84 |
| PRZEMYŚL | -3,76* | -1,51 | 0,71 | -9,03 | -0,65 | 0,30 |
| PUŁAWY | -3,15 | -2,25 | -5,46 | 0,43 | -0,01 | 2,88 |
| RADOM | -0,77 | -1,86 | 0,43 | -3,19 | 1,89* | 3,58 |
| RYBNIK | -0,29 | -2,61 | 1,31 | -6,08* | 1,05 | 2,32 |
| RZESZÓW | -2,22 | -2,83* | -3,16 | -7,81* | 2,43 | -2,46 |
| SANDOMIERZ & JĘDRZEJÓW | -2,48 | -4,78** | -0,51 | -5,19 | -0,27 | -0,97 |
| SIEDLCE | -2,45** | -1,07 | 2,04 | -5,02* | -0,21 | -4,32 |
| SIERADZ | 0,02 | -3,70 | -7,28* | -7,75 | -0,23 | -0,77 |
| SKIERNIEWICE | 1,83 | -2,82* | -3,26 | -3,97* | 0,53 | 2,14 |
| SŁUPSK | -1,90 | -2,35 | -0,27 | -9,28** | -1,77 | -9,22* |
| SOSNOWIEC | -2,55* | -7,90*** | -10,55 | -2,51 | -0,20 | 1,27 |
| STAROGARD GDAŃSKI | -3,75* | -9,30** | -3,70 | -4,92 | -3,31* | 3,29 |
| SUWAŁKI | -2,00 | -2,47* | 0,57 | 1,33 | -0,47 | 3,24 |
| CITY OF SZCZECIN | -2,40 | -13,55* | -8,29* | -9,37* | 0,23 | -1,08 |
| SZCZECIN (RURAL) | -1,72 | -6,04** | -3,74 | -8,53** | 0,16 | 0,61 |
| SZCZECINEK & PYRZYCE | 3.14* | -5.47* | -0.76 | -5.32 | -0.08 | 0.75 |
| ŚWIECKO | -0,20 | -0,43 | -3,00 | 1,05 | 2,12 | -1,90 |
| TARNOBRZEG | -1,89 | -1,55 | -4,41* | -6,79* | 2,11 | -1,68 |
| TARNÓW | 0,21 | -3,46 | 0,29 | -6,18** | -1,84 | -4,02 |
| TRICITY | -2,33 | -5,52** | -6,02 | -3,34 | -0,58 | 2,35 |
| TYCHY | -0,98 | -3,64* | -1,55 | -7,74 | 0,35 | 1,10 |
| WAŁBRZYCH | -2,58* | -3,49* | -1,69 | -4,81 | 0,80 | 4,66* |

*** - p < 0.05 ** - p < 0.01 *** - p < 0.001**

| **Subregion** | **C18-C21** | **C34** | **C44** | **C64** | **C50** | **C61** |
| --- | --- | --- | --- | --- | --- | --- |
| WARSAW | -2,85 | -2,13 | -5,65 | -7,18** | 0,15 | -2,75 |
| WARSAW EAST | -1,39 | -3,12** | -1,26 | -4,94 | 1,23 | -0,85 |
| WARSAW WEST | -1,79 | -2,88* | -5,24 | -5,84** | 2,39* | -1,91 |
| WŁOCŁAWEK | -3,54 | -3,26* | -3,78 | 1,00 | -0,03 | 1,68 |
| CITY OF WROCŁAW | -2,08 | -5,39* | -0,04 | -11,20** | 1,25 | 0,95 |
| WROCŁAW (RURAL) | -0,30 | -2,24** | 3,77 | -9,15** | 0,68 | 3,01 |
| ZIELONA GÓRA | -1,99* | -2,84* | -5,54 | -1,56 | -0,13 | 0,92 |
| ŻYRARDÓW | -1,16 | -3,69* | -6,20 | -6,70 | -1,84 | 0,09 |

*** - p < 0.05 ** - p < 0.01 *** - p < 0.001**
